# Fluorescent Labeling of Peroxisome and Nuclear in *Colletotrichum aenigma*

**DOI:** 10.3390/jof9040493

**Published:** 2023-04-21

**Authors:** Shendan Yu, Jing Wang, Rongyao Chai, Haiping Qiu, Ziqi Lu, Zhen Zhang, Lin Li, Jiaoyu Wang, Guochang Sun

**Affiliations:** 1 College of Life Sciences, Zhejiang Normal University, Jinhua 321004, China; yushendan6@163.com; 2 State Key Laboratory for Managing Biotic and Chemical Threats to the Quality and Safety of Agro-Products, Key Laboratory of Biotechnology in Plant Protection of MOA of China and Zhejiang Province, Institute of Plant Protection and Microbiology, Zhejiang Academy of Agricultural Sciences, Hangzhou 310021, China; wj9311@163.com (J.W.); rychai@sina.com (R.C.); qiuhping@163.com (H.Q.); zzhangcn928@sina.com (Z.Z.); 11816015@zju.edu.cn (L.L.); 3 College of Advanced Agricultural Sciences, Zhejiang Agriculture and Forestry University, Hangzhou 310007, China; luziqi18234559092@163.com

**Keywords:** *Colletotrichum aenigma*, fluorescent protein labeling, peroxisome, nucleus

## Abstract

Anthracnose is one of the most widespread and destructive diseases in grapes. Grape anthracnose can be caused by various *Colletotrichum* species, such as *Colletotrichum gloeosporioides* and *Colletotrichum cuspidosporium*. In recent years, *Colletotrichum aenigma* was reported as a causal agent of Grape anthracnose in China and South Korea. Peroxisome is an important organelle in eukaryotes, which plays a very important role in the growth, development, and pathogenicity of several plant-pathogenic fungal species i, but it has not been reported in *C. aenigma.* In this work, the peroxisome of *C. aenigma* was labeled with a fluorescent protein, using green fluorescent protein (GFP) and red fluorescent protein (DsRED and mCherry) as reporter genes. Via *Agrobacterium tumefaciens*-mediated transformation (*At*MT), two fluorescent fusion vectors to mark the peroxisomes, with GFP and DsRED, respectively, were introduced into a wild-type strain of *C. aenigma*. In the transformants, bright dots of green or red fluorescence in hyphae and spores could be seen in the strains labeled peroxisome. The nuclei labeled by the same method showed bright round fluorescent spots. In addition, we also combined fluorescent protein labeling with chemical staining to show the localization more clearly. The ideal peroxisome and nuclear fluorescence-labeled *C. aenigma strain* was obtained, which provided a reference for the study of its growth, development, and pathogenicity.

## 1. Introduction

China is the largest producer and consumer of fresh grapes in the world. There are reports indicating that after conducting field investigations in the main grape-producing areas of Xinjiang and Hebei, it was found that the grape-planting area of China has remained stable at over 10 million mu over the past five years. The production of grapes in China continued to increase, reaching 13.667 million tons in 2018 and becoming the world’s largest producer. The distribution of grape-planting areas in China is concentrated, with the main production areas located in historically advantageous planting areas, such as Xinjiang, Shanxi, Hebei, and Shandong. The planting areas of five provinces have reached 4.629 million mu, accounting for 42.6% of the total area.

Grape anthracnose, also known as late rot, is an important disease impacting grapes that is mainly caused by the fungus *Colletotrichum* species, such as *Colletotrichum gloeosporioides* and *Colletotrichum cuspidosporium* [1]. The disease often occurs at the end of grape growing and fruit ripening, which mainly harms fruits and ear rachis of grapes. In addition, it can also damage leaves, new shoots, tendrils, fruit stalks, and other parts. The diseased fruits and leaves generate black-brown anthracnose spots, and the fruit surface is often festered with extravasated juice, which seriously reduces the yield and quality of the grapes. At present, the disease occurs in most grape-producing areas in China, and in recent years, *Colletotrichum aenigma* was also reported as a causal agent of Grape anthracnose in China and South Korea [2,3,4,5].

Peroxisome is a kind of monolayer-coated organelle with active metabolisms and highly dynamic changes in eukaryotes, which is mainly involved in the metabolism of fat, the production and scavenging of reactive oxygen species, etc. Additionally, it has been reported that peroxisome is essential for the pathogenicity of many phytopathogenic fungi, including *Magnaporthe oryzae*, *Botrytis cinerea*, *Fusarium graminearum*, and so on [6]. However, it is still undocumented whether the peroxisomes function in the infection cycles of *C. aenigma*. The peroxisomes were found to be induced and greatly proliferated at the early stages of the conidial germination and infection of *M. orzyae* [7]. Therefore, the study of peroxisome distribution and dynamics in the process of growth, development, and pathogenesis is of great significance in revealing the involvement of peroxisomes in the pathogenicity of phytopathogenic fungi, including *C. aenigma*.

Fluorescent protein labeling is an effective method of studying promoter activity, gene expression dynamics, cellular and subcellular localization of proteins, and the growth and development of organisms [8]. Green fluorescent protein (GFP), first found in *Aequorea victoria* in 1962, has stable, intuitive, and convenient fluorescence properties and is the most widely used fluorescent protein at present. Red fluorescent protein (RFP) was isolated from *Discosoma* sp. by MATZ et al. in 1999. The different variants of red fluorescent protein show different spectral and physicochemical properties, emitting various colors of fluorescence. For example, mCherry emits 610 nm fluorescence under 587 nm excitation light and cherry-red under a fluorescence microscope. It has the advantages of rapid ripening and good monomer properties and has a wide range of applications. Calcofluor white (CFW) and DAPI are two kinds of dyes that display blue fluorescence under a fluorescent microscope [9]. CFW can bind cellulose and chitin in the fungal cell wall and is thus widely used for labeling cell walls in fungi. DAPI is a nuclei dye that penetrates the cell membrane and binds specifically to DNA in a non-embedded way [10]. Histone is a basic DNA-binding protein, which is mainly located in the nucleus. The fusion expression of histone and fluorescent protein can realize the fluorescent labeling of the nucleus and assist in the dynamic observation of the nucleus [11]. Wang J Y et al. fused the peroxisome localization signal PTS1/PTS2 into GFP, respectively. They then successfully located the peroxisome of *Magnaporthe oryzae* [12]. Fluorescent protein localization was also utilized in *Fusarium oxysporum f. sp. niveum* [13] and *Botrytis cinerea* [6]. We can also study the function of genes and the dynamic changes of nuclei in the process of infection through the location of the peroxisome and the nuclei. However, there is no study on the fluorescence localization of peroxisome in *C. aenigma.*

In this present work, GFP-PTS1 [13], DsRED-PTS1 [13], mCherry-H2B [14], and PNMCherryA, under different promoters, were used as fluorescent markers to label the peroxisomes and nuclei in *C. aenigma* via *Agrobacterium tumefaciens*-mediated fungal transformation (*At*MT). Using the fluorescently labeled strains, the peroxisomal dynamics and nuclear distribution in *C. aenigma* were detected under a fluorescent microscope. In addition, we obtained a library of T-DNA insertion transformants for mutant selection. Consequently, we provide a tool and basic data for the further study of the organelle biogenesis, fungal growth, development, and pathogenicity of *C. aenigma*.

## 2. Materials and Methods

### 2.1. Test Strains and Culture Conditions

*C. aenigma* and its transformants were cultured on the complete medium (CM) [15] in 28 °C darkness. *Colletotrichum aenigma* (Genbank Taxonomy ID: 1215731) strain NH-8 was isolated from a grape vineyard in Ninghai country, Zhejiang province, China, identified by the morphology and the sequences of ITS, GAPDH, and tubulin, and kept in the fungal strain store in Zhejiang Academy of Agricultural Sciences (accession number ZN-05827).

### 2.2. Plasmids and AtMT

PHMGA, pHMR1 [16], and pKD9-mCherry-H2B [17] were introduced into *C. aenigma* by AtMT [18] transformation, and the transformants CA-HMGA, CA-HMR1, and CA-mCherry-H2B were obtained. In the same way, PNMCherryA was introduced into CA-HMGA to obtain a CA-HMGA-PNMCherryA colocalized strain. *A. tumefaciens* strains were cultured in a YEB liquid medium containing 50 μg/mL ampicillin, 50 μg/mL rifampicin, and 50 μg/mL kanamycin in 28 °C darkness for 2 days with 180 r/min [6]. Obtaining *C. aenigma* spores which were cultured on CM medium at 28 °C for 5 days, we then prepared the spore suspension into 1 × 10^6^/m L. Afterward, the spore liquid and *A. tumefaciens* were co-cultured on IM medium [19] at 23 °C for 2 days. Finally, the transformants were screened on a CM medium plate containing 150 μg/mL hygromycin B.

### 2.3. Fluorescence Stability and PCR Detection of Transformants

**Figure 1:**
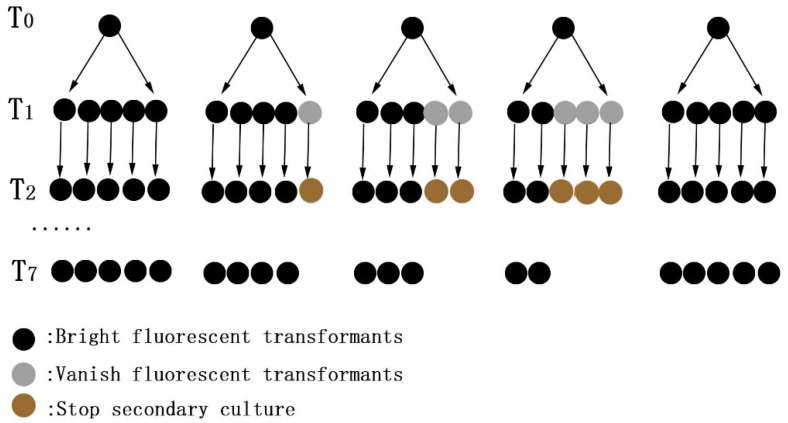
Stability of the fluorescent transformants of CA-HMR1. Five transformants (T0) with bright fluorescence expression were selected randomly by resistance screening and fluorescence microscopy. Sub-cultured to generate progeny transformants (T1), until the T7 generation. The fluorescence of each transformant was observed to analyze the fluorescence stability of the transformants.

Taking CA-HMR1 transformants as an example to detect genetic stability is shown in Figure 1. Five CA-HMR1 transformants (T0) with bright fluorescence expression were selected randomly by resistance screening and fluorescence microscopy. Five mycelial blocks were taken from the colonial edges of each of the T0 transformants and subcultured to generate 25 progeny transformants (T1). The fluorescence of each T1 transformant was observed and those with fluorescence were sub-cultured to obtain T2 generation, successively, until the T7 generation. The fluorescence of hyphae and the spores of all the progeny transformants was observed by a fluorescence microscope to analyze the fluorescence stability of the transformants.

The transformants of each generation were detected by PCR to determine whether they contained the hygromycin resistance gene and the corresponding fluorescent protein gene. Genomic DNA was extracted by the CTAB [20] method. The fragments of hygromycin, GFP, DsRED, mCherry, and G418 genes were amplified by using the primer pairs HPH52/HPH34, GFP1-CHK1/GFP-CHK2, RED1/RED2, RED3/RED4, and NEO1/NEO2, respectively. The genomic DNA of the *C. aenigma* wild strain was used as a negative control, and the corresponding plasmid DNA was used as a positive control. The PCR products were detected by electrophoresis with 1.0% agarose gel under 150V for 20 min. The primers used were listed in Table S1 [7] and PCR procedures were attached in Table S2.

### 2.4. Laser Confocal Observation and Co-Localization with Fluorescent Dyes

The transformants with strong fluorescence expression, clear localization, and stable subculture were selected, and a small number of hyphae and spores were selected to observe and record the fluorescence localization using a laser confocal microscope (ZEISS LSM780).

The red fluorescence was observed at an excitation wavelength of 543 nm and an emission wavelength of 570–630 nm. The green fluorescence was observed at an excitation wavelength of 488 nm and an emission wavelength of 495–550 nm. In addition, the blue fluorescence was observed at an excitation wavelength of 405 nm and an emission wavelength of 410–480 nm.

CFW and DAPI Staining: 10 μL 10 μg/mL CFW staining solution was added to avoid light staining for 5 min, and the blue fluorescence produced by the cell wall was observed [8]. Using a similar method, 10 μL 50 μg/mL DAPI staining solution was added to observe the blue fluorescence produced by the nucleus [21].

### 2.5. Comparison of Phenotype and Spore Production of Transformants

The tested strain was inoculated on the CM medium plate for 3 days, and then the round mycelial blocks with a diameter of 0.5 cm were obtained at the colony edge of the transformant and strain *C. aenigma* with a hole punch, and finally transferred to the new CM medium plate with 3 repeats for each strain. After 3 days of dark culture at 28 °C, the colony diameter was measured and photographed, and the difference in growth rates was statistically analyzed. The growth rate was calculated with V = (D − d)/T; V is the growth rate (mm/d); D is the average diameter of the colony (mm); d is the diameter of the inoculated plaque (mm); and T is the growth time (d).

To harvest the spores, 4 mL of sterilized distilled water was added to each CM culture plate. Then the colony surface was gently scraped with a disposable coating stick to wash the spores into the water. The spore suspension was filtered with three layers of sterilized lens wipes with 3 repeats for each treatment. The spore concentration was calculated on a Hemocytometer, and the spore generation of each strain was calculated and compared.

### 2.6. Pathogenicity Test

The pathogenicity was tested by acupuncture inoculation (acupuncture 0.5 cm). The spore suspensions of the transformants and wild strain of *C. aenigma* were prepared in 10^6^ mL^−1^. Afterward, 20 fresh grapes and a few leaves were wiped briefly with 75% alcohol, naturally dried, and acupunctured using sterile needles, then they were inoculated by dropping the suspension on the acupunctured sites with pipettes.

The mycelial blocks were also used in inoculation by placing them on the acupuncture site of the leaves. The sterile water was used as the control, and a group of non-invasive inoculation groups was set up. The treatment was repeated 3 times in each group. After inoculation, the fruits were cultured in the dark at 28 °C and we added 10 mL of sterile water with sterilized cotton or filter paper to maintain humidity. The incidence of fruits and leaves was observed and recorded.

## 3. Results

### 3.1. Screening and Genetic Stability of Transformants

**Figure 2:**
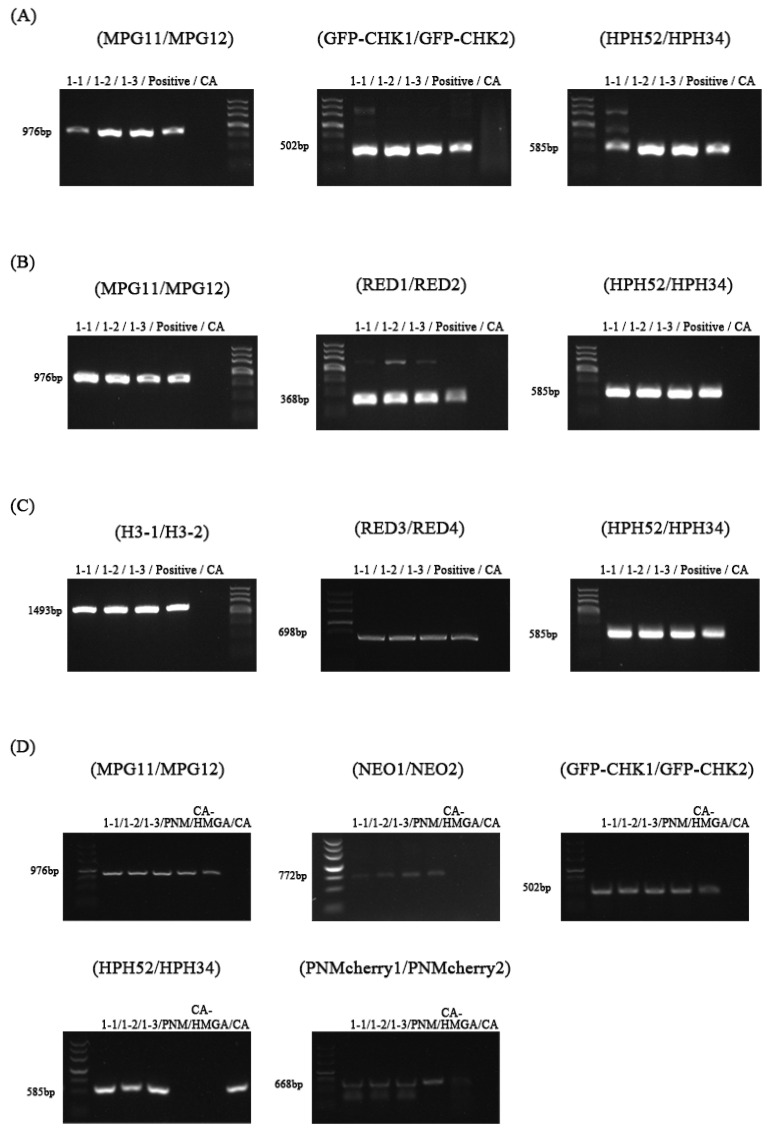
Confirmation of the fluorescent transformants using PCR amplification. The randomly selected CA-HMGA ((**A**): 1-1–1-3), CA-HMR1 ((**B**): 2-1–2-3), CA-mCherry-H2B ((**C**): 3-1–3-3), and CA-HMGA-PNMcherryA ((**D**): 2-1–2-3) transformants, three for each, were checked by PCR amplification with the primer pairs HPH52/HPH34, GFPCHK1/GFPCHK2, RED3/RED4, RED1/RED2, and PNMcherryA1/PNMcherryA2 to detect HPH, GFP, mCherry, RFP, and PNMcherryA fragments, respectively. CA: the wild-type strain; positive: the positive controls with the plasmids pHMGA, pHMR1, pKD9-mCherry-H2B, PNM, and CA-HMGA as templates; marker III.

The partial transformants of CA-HMGA (Figure 2A), CA-HMR1(Figure 2B), CA-mCherry-H2B (Figure 2C), and CA-HMGA-PNMcherryA (Figure 2D) that gave off corresponding fluorescence were randomly selected for PCR verification. The results showed that the corresponding resistance gene fragment and fluorescent marker gene fragment could be amplified from all of the inverters. The size of each fragment was the same as that of the corresponding positive control, but there was no amplified band in the wild-type control. The results showed that both the resistance gene and the fluorescent marker gene had been successfully integrated into the detected inverters.

In order to determine the genetic stability of transformants, five CA-HMR1 transformants (T0 generation) expressing high green fluorescence were randomly selected and subcultured for 7 generations, and the fluorescence of each generation was observed. The results showed that among the 25 transformants of T1 generation, 6 transformants lost fluorescence. In the T2 generation, 19 transformants could emit light, and 6 of 25 transformants disappeared after subculture to the T7 generation. However, for the transformants whose fluorescence disappeared, the fluorescence could not be recovered after 2 generations. The results showed that the subculture led to the loss of fluorescence of some of the transformants. The genetic instability of *C. aenigma* transformants may be related to their multinucleated characteristics. The cells of transformants with T-DNA integrations in partial nuclei can emit red fluorescence. However, in the process of subculture, the untransformed nuclei occasionally replaced and occupied the cells, and resulted in the loss of fluorescence. Therefore, the stable strains can be obtained via successive subcultures and selection.

### 3.2. Growth, Sporulation, and Pathogenicity of Transformants

**Figure 3:**
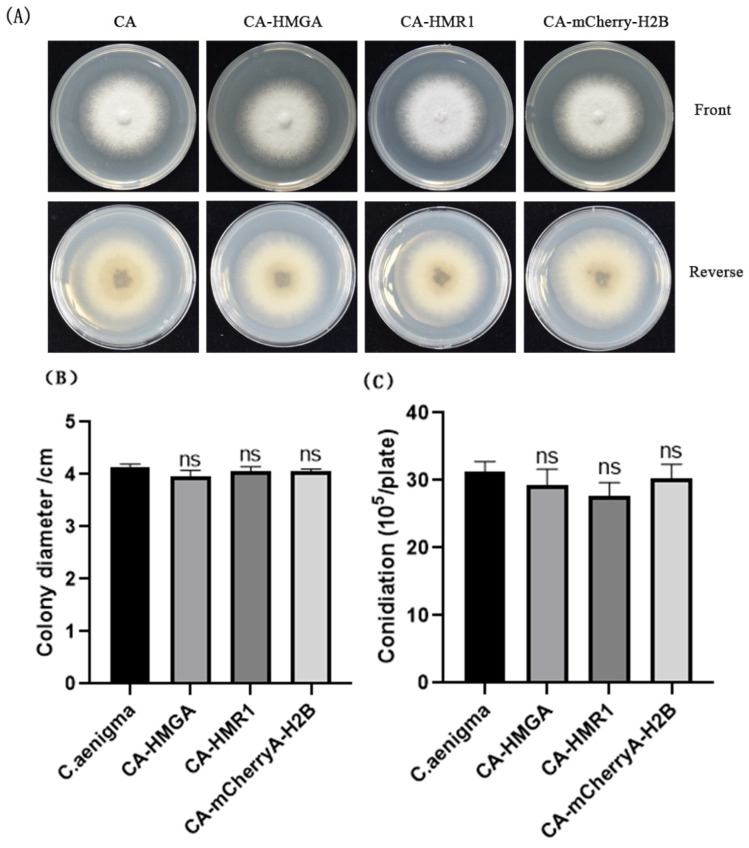
Colonial morphology, radial growth, and conidiation of the transformants. (**A**) colonies of the *C. aenigma* wide type and transformants cultured on CM for 3 days; (**B**) colonial dimeters of the strains cultured on CM for 3 days; and (**C**) conidiation of the strains cultured on CM for 3 days. ns: no significant difference between transformants and *C. aenigma*.

Through transformation, a random insertion transformation library of *C. aenigma* was obtained at the same time as the fluorescence localization. In order to test the effect of foreign gene introduction on the phenotype of transformants, 5 transformants of CA-HMGA, CA-HMR1, and CA-mCherry-H2B were randomly selected to observe the colony morphology (Figure 3A), spore production (Figure 3C), growth rate (Figure 3B), and pathogenicity. The results showed that the colony morphology, growth rate, spore production, and pathogenicity of most of the transformants were not significantly different from those of *C. aenigma*, and a few transformants showed slower growth rates. As a result, we concluded that the phenotypic variation of transformants caused by the random insertion of foreign genes is a low-probability event, and most fluorescent transformants can simulate the dynamics of organelles in the wild type. At the same time, specific phenotypic transformants can be obtained through screening, which lays a foundation for the identification and study of functional genes.

**Figure 4:**
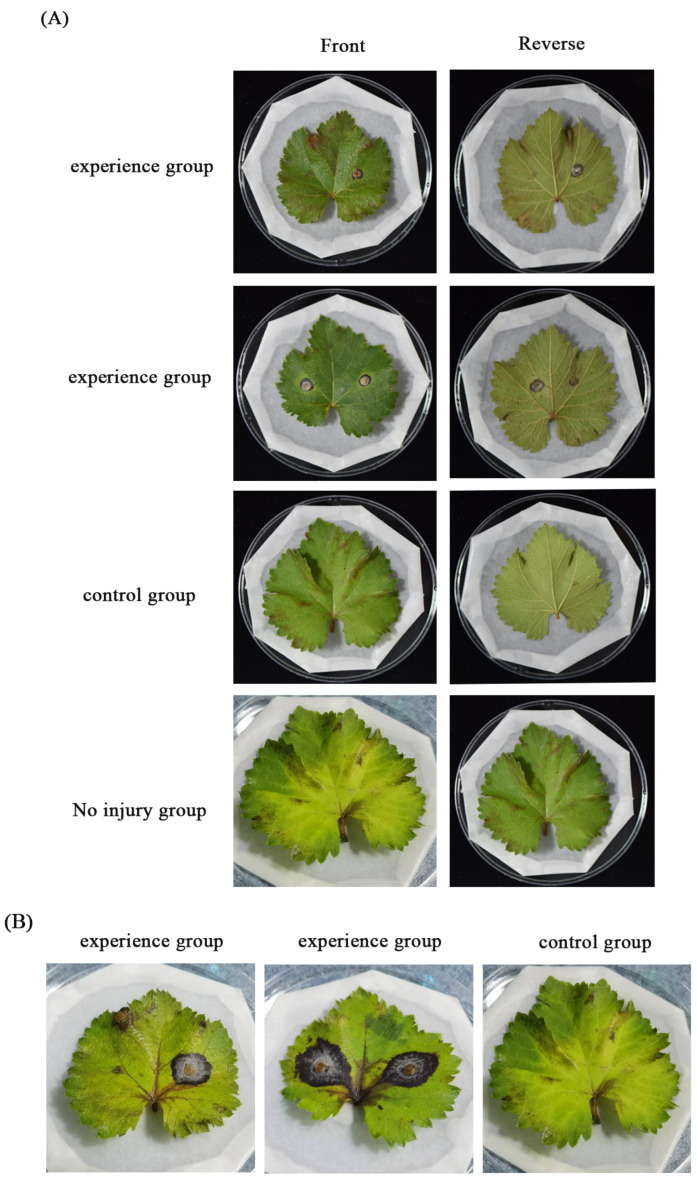

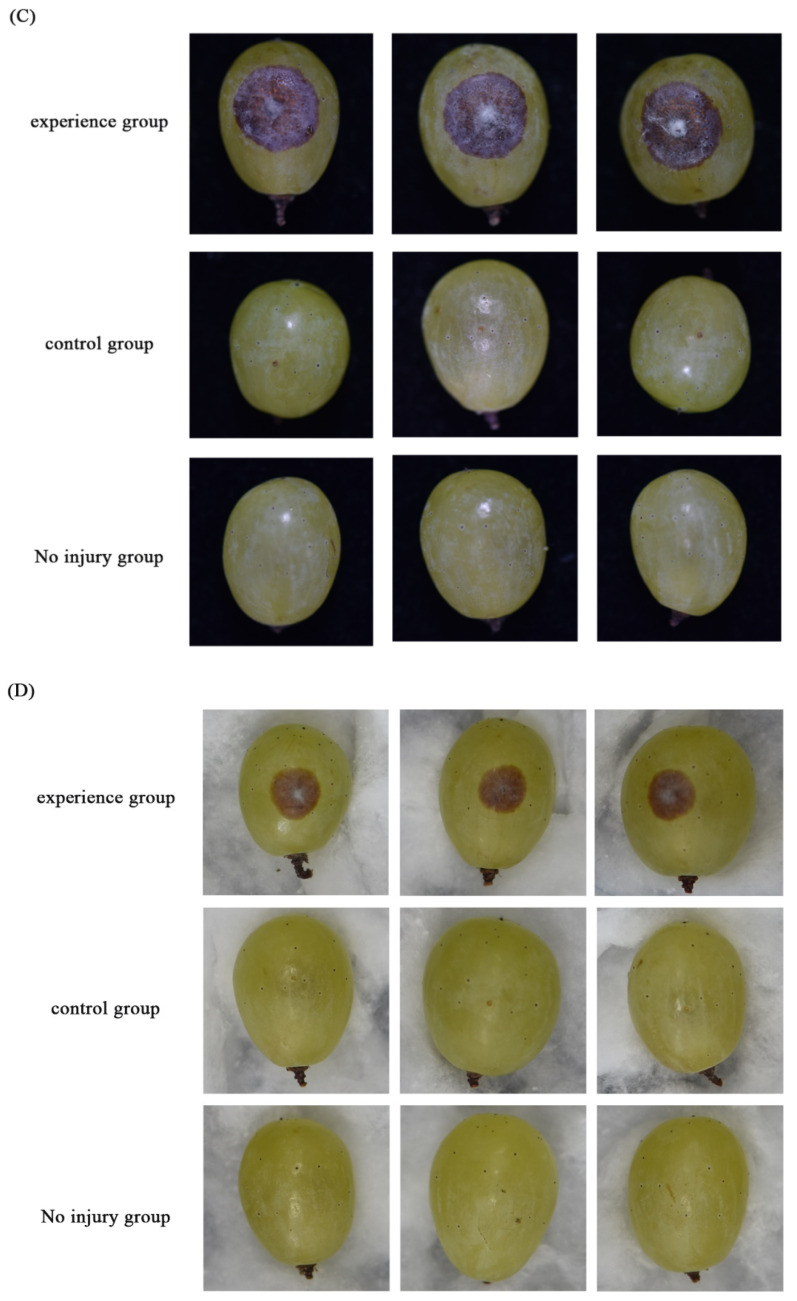
Pathogenicity of the transformants on grape leaves and fruits. (**A**) The grape leaves were inoculated with mycelial blocks and the symptoms were checked after 3 days and seven days (**B**); (**C**) Grape fruits were inoculated with spore suspension and checked after 3 days and 6 days (**D**).

Secondly, several strains of *C. aenigma*, CA-HMGA, and CA-mCherry-H2B were selected to test virulence. After inoculating healthy grape leaves for three days, the inoculation site began to turn black (Figure 4A). Seven days later, the brown became darker and darker, the lesion area also expanded continuously (Figure 4B), and it began showing the symptoms of grape anthracnose. Brown disease spots also appeared in the fruit three days after inoculation (Figure 4D) and the disease became more serious seven days later (Figure 4C). At the same time, all of the leaves and fruits inoculated with spore suspension or mycelial blocks showed the above symptoms. While the grape leaves in the control group and the non-injury inoculation group had no susceptible symptoms, these results show that the random insertion of foreign genes will not make the pathogenicity of *C. aenigma* disappear.

### 3.3. Fluorescence Localization of Nucleus

**Figure 5:**
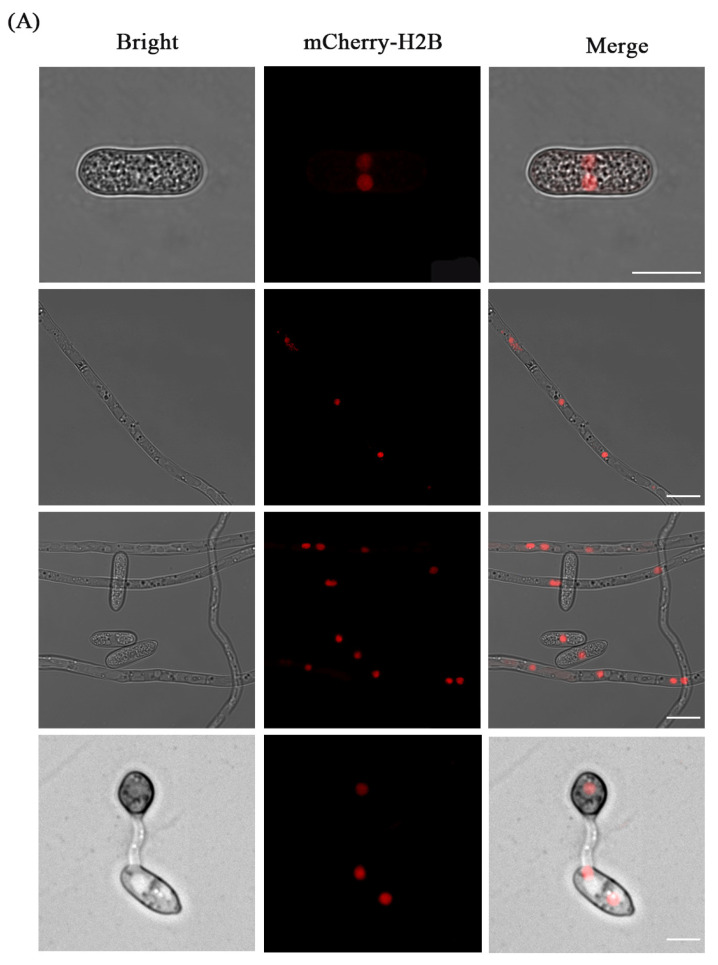

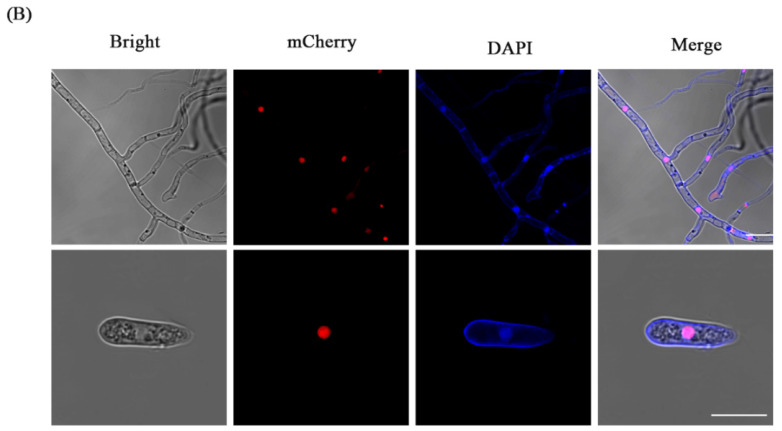
Fluorescent labeling of the nuclei in *C. aenigma* with mCherry-H2B. Bars = 10 μm. (**A**) The mCherry-H2B transformants were detected under a confocal microscope to check the labeled nuclei in spores, hyphae, and appressoria; (**B**) The co-localization of mCherry-H2B and DAPI in the nuclei of the transformants.

The successfully verified transformants were selected to observe the fluorescence expression and localization under confocal fluorescence microscopy. Under the action of the H3 promoter, mCherry-H2B fusion protein showed a high brightness of red fluorescence in both mycelium and spores. The red fluorescence was concentrated on the round spots inside the cells, and the location and size of the spots were consistent with the characteristics of nuclear localization. We also found that with the advance of spore germination, the nucleus in the spore gradually disappeared, while new nuclei appeared in the newly formed appressorium (Figure 5A). These dots overlapped with the blue fluorescence formed by nuclear DAPI staining, indicating that the fluorescence was correctly located in the nucleus. Both mycelial cells and spore cells contained 1-2 nuclei (Figure 5B).

### 3.4. Fluorescence Labeling of Peroxisome

**Figure 6:**
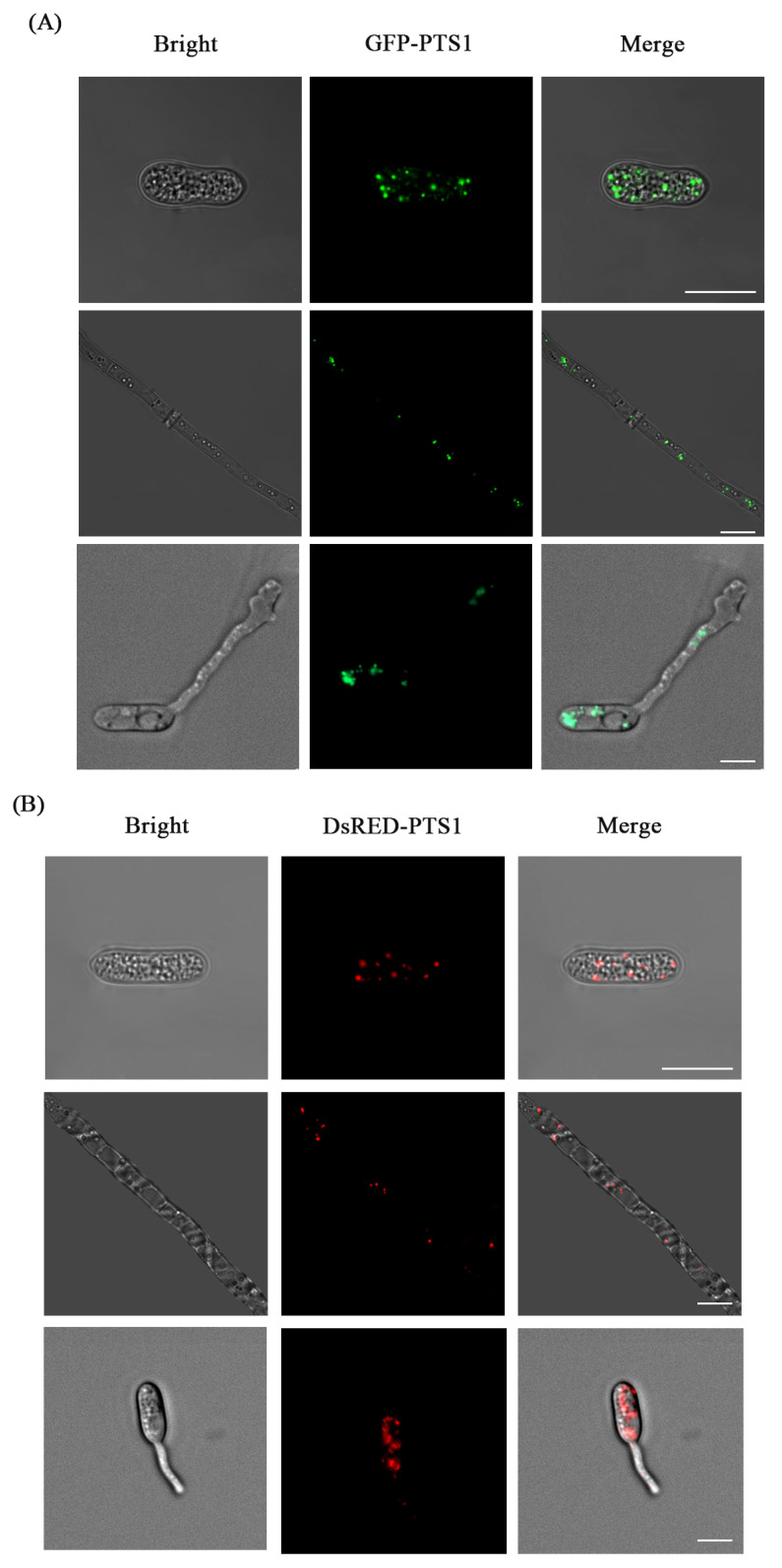

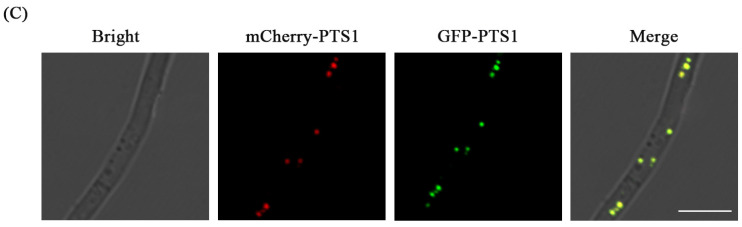
Fluorescent labeling of the peroxisomes in *C. aenigma* with GFP-PTS1, DsRED-PTS1, and mCherry-PTS1. Bars = 10 μm. (**A**) GFP-PTS1 labeled peroxisomes in hyphae and spores; (**B**) DsRED-PTS1 labeled peroxisomes in hyphae and spores; and (**C**) co-localization of mCherry-PTS1 and GFP-PTS1 labeled peroxisomes in hyphae.

Under a laser confocal microscope, the transformants of CA-HMGA, CA-HMR1, and CA-HMGA-PNMcherry were observed. Under the action of the *MPG1* gene promoter, the green fluorescence of GFP-PTS1(Figure 6A), red fluorescence (Figure 6B), or red-green fluorescence (Figure 6C) of DsRED-PTS1 were observed in spores and mycelium cells. The fluorescence was distributed in the interior of the cell in small dots with a diameter of 0.2–1.0 μm, which accorded with the size and distribution characteristics of fungal peroxisome. The results showed that GFP-PTS1 and DsRED-PTS1 were located correctly on the peroxisomes of *C. aenigma*. We can also clearly see the green fluorescence and red fluorescence localization in the appressorium. Peroxisome slowly appears in the germ tube over time, so we can observe the number and dynamic changes of peroxisome.

### 3.5. The CFW Staining of the Fluorescent Transformants

**Figure 7:**
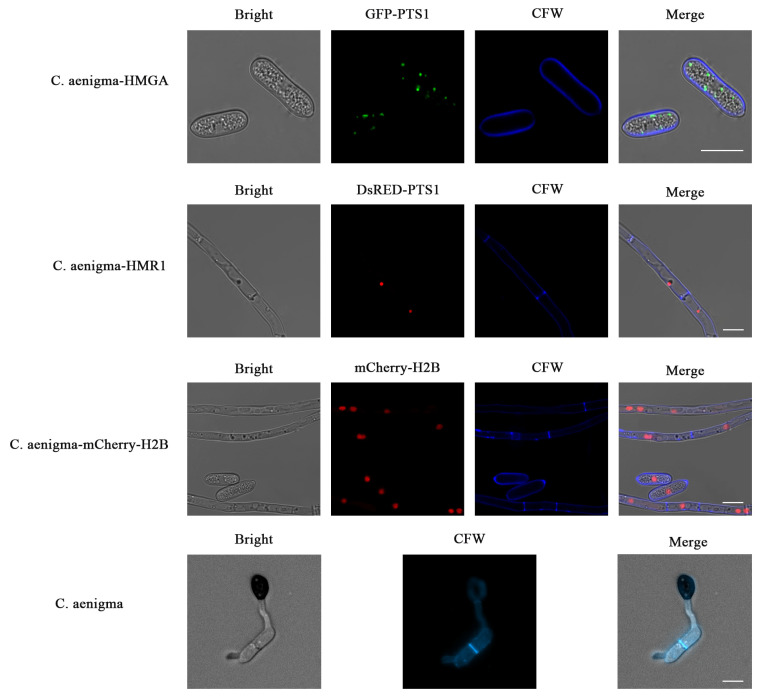
The calcofluor white (CFW) staining of the fluorescent transformants, Bars = 10 μm. The CA-HMGA, CA-HMR1, and CA-mCherry-H2B transformants and the wild type of *C. aenigma* were stained with CFW and detected under the confocal microscope. The cell walls stained by the CFW emitted the blue fluorescence, well co-existed with the green fluorescence of GFP-PTS1 and the red fluorescence of DsRED-PTS1 and mCherry-H2B.

In order to observe the location of different cell structures and organelles more clearly and intuitively, the appressorium of *C. aenigma* and the cell walls of the three transformants CA-HMGA, CA-HMR1, and CA-mCherry-H2B were stained with CFW for co-localization observation (Figure 7). As observed under a confocal microscope, the blue fluorescence emitted by CFW staining had no obvious interference with the red or green fluorescence emitted by fluorescent proteins. Blue fluorescence is located in the cell wall of hyphae or spores, clearly outlining the cell, while green or red fluorescence is located in the corresponding position of the cell. It shows an ideal localization effect, which further confirms the correct localization of fluorescent proteins in the peroxisome and nucleus.

## 4. Discussion

Using fluorescent protein labeling to track the growth, development, and pathogenicity of fungal strains is an effective method in plant and animal pathogenic fungi. At the same time, fluorescent proteins are also widely used to study the spatial-temporal expression of genes, and the cellular and subcellular localization of proteins. At present, many different fluorescent proteins have been applied in fungi, such as *Neurospora crassa*, *M. oryzae*, and so on [21]. However, compared with fungal species, such as *M. oryzae* and *F. graminearum*, there are fewer studies on the molecular mechanism of the *C. aenigma* infection and the labeling of organelles with fluorescent proteins. In this study, the peroxisome and nucleus of *C. aenigma* were successfully labeled by GFP and the red fluorescent proteins DsRED and mCherry.

The number of nuclei and the dynamic processes of nuclear division, movement, and degradation are some of the important contents of fungal developmental biology [22].

In the process of the appressorium formation of *M. oryzae*, there is a precise regulation of nuclear division and distribution to daughter cells. As a multinucleated fungus, the mechanism of nuclear division, separation, and regulation of *C. aenigma* may be more complex. The formation and biochemical metabolism of peroxisome are essential to the infection of *M. oryzae* and *B. cinerea* [23]. The fluorescent transformants obtained in this study have high abundance expression, high fluorescence brightness, and good dispersion, and can accurately trace the location, size, and movement of the nucleus and peroxisome in *C. aenigma*, which provides a reference and source of material for studying the growth, development, pathogenic process, and molecular mechanism of the fungus.

The appropriate promoter to trigger the expression of the fluorescent proteins is the basis of fluorescent labeling. The results showed that *H3* and *MPG1* promoters from *M. oryzae* could be used to trigger the gene expression in *C. aenigma*. The fluorescent protein genes under the two promoters were abundantly expressed in the cells of *C. aenigma* in hyphae and conidia. The expression intensity was adequate for labeling the peroxisomes and the nuclei. Our results thus gave more options for effective fungal promoters regarding the study of fungal genes. In order to show the localization more clearly, we also combined applying the localization of fluorescent proteins with chemical fluorescent staining. We provided important data for the study of cell structure, gene function, and protein-protein interaction.

The transformation efficiency of *C. aenigma* is comparable to that of filamentous fungi, such as *M. oryzae* and *F. graminearum*, but the genetic stability of the transformants of *C. aenigma* is lower, which may be due to the multinucleation of *C. aenigma*. In addition, the colony morphology, growth rate, sporulation, and pathogenicity in most of the transformants were not significantly different from those of the wild type. The results indicated that the growth and development of most of the transformants were not affected by the transformation processes, or the expression of hygromycin and fluorescent proteins.

Therefore, most of the fluorescent strains would be capable of tracing the organelles in further cellular research. A very small portion of the transformants showed phenotypic variation, in which the T-DNA may have been inserted into some important gene loci. These transformants were important in identifying the functional genes in fungal molecular biology. Thus, our work provides a useful tool for the study of molecular and cellular biology in fungi.

## Data Availability

Not applicable.

## Supplementary Materials

The following supporting information can be downloaded at: https://www.mdpi.com/article/10.3390/jof9040493/s1, Table S1: Primers used in this work. Table S2: PCR amplification information.
