# Supplementary material for: Fluorescent Labeling of Peroxisome and Nuclear in Colletotrichum aenigma
Source: J Fungi (Basel). 2023 Apr 21;9(4):493. doi: 10.3390/jof9040493 (PMC10142913; doi:10.3390/jof9040493)
Supplement: Supplementary file 1 [file jof-09-00493-s001.zip › jof-2327359-supplementary.pdf]

Table S1. Primers used in this work.

Names	Sequences	Amplicons and lengths
HPH52	5′-AGCTGCGCCGATGGTTTCTACAA-3′	HPH gene fragment, 585 bp
HPH34	5′-GCGCGTCTGCTGCTCCATACAA-3′	
MPG11	5′-TCACGACTGGGAGTAGAAAGA-3′	Fragment of MPG1 gene promoter, 976 bp
MPG12	5′-CCAGATTCCAGGGTTGCTAAA-3′	
GFP-CHK1	5′-GCCACCTACGGCAAGCTGACCCTG-3′	GFP gene fragment, 502 bp
GFP-CHK2	5′-GGGTGCTCAGGTAGTGGTTGTCGG-3′	
RED1	5′-GATGGTGTAGTCCTCGTTGTG-3′	DsRed gene fragment, 368 bp
RED2	5′-GACTACTTGAAGCTGTCCTTCC-3′	
RED3	5′-CCGGGGTGAGCAAGGGCGAGG-3′	mCherry gene fragment, 698 bp
RED4	5′-TCTAGAGCCGCCGGTGGAGTG-3′	
NEO1	5′-CGGGTAGCCAACGCTATGTCC-3′	NEO gene fragment, 772 bp
NEO2	5′-GGACGACCCGGTCATACCTTC-3′	
H3-1	5′-AGTCATGTTGATTGAGGTGT-3′	Fragment of H3 gene promoter, 1493 bp
H3-2	5′GGCCATTGTGATTGATTTGTG-3′	
PNMcherryA1	5′TCGGCGCGTTCGTACTGTTCC-3′	PNMcherryA gene fragment, 668bp
PNMcherryA2	5′ATGGTGAGCAAGGGCGAGGAG-3′	

Table S2. PCR amplification information

Names	Reaction procedure
HPH52<br>HPH34	95℃−Pre-denaturation 3 min, 95℃-Denaturation 15s, 60℃−Annealing 15s, 72℃-Extend 90s−30 cycles, 72 ℃ extended 5 min, 4℃-for preservation.
MPG11<br>MPG12	95℃−Pre-denaturation 3 min, 95℃-Denaturation 15s, 56℃−Annealing 15s, 72℃-Extend 90s−30 cycles, 72 ℃ extended 5 min, 4℃-for preservation.
GFP-CHK1<br>GFP-CHK2	95℃−Pre-denaturation 3 min, 95℃-Denaturation 15s, 60℃−Annealing 15s, 72℃-Extend 90s−30 cycles, 72 ℃ extended 5 min, 4℃-for preservation.
RED1<br>RED2	95℃−Pre-denaturation 3 min, 95℃-Denaturation 15s, 56℃−Annealing 15s, 72℃-Extend 90s−30 cycles, 72 ℃ extended 5 min, 4℃-for preservation.
RED3<br>RED4	95℃−Pre-denaturation 3 min, 95℃-Denaturation 15s, 61℃−Annealing 15s, 72℃-Extend 90s−30 cycles, 72 ℃ extended 5 min, 4℃-for preservation.
NEO1<br>NEO2	95℃−Pre-denaturation 3 min, 95℃-Denaturation 15s, 58℃−Annealing 15s, 72℃-Extend 90s−30 cycles, 72 ℃ extended 5 min, 4℃-for preservation.
H3-1<br>H3-2	95℃−Pre-denaturation 3 min, 95℃-Denaturation 15s, 53℃−Annealing 15s, 72℃-Extend 90s−30 cycles, 72 ℃ extended 5 min, 4℃-for preservation.
PNMcherryA1<br>PNMcherryA2	95℃−Pre-denaturation 3 min, 95℃-Denaturation 15s, 58℃−Annealing 15s, 72℃-Extend 90s−30 cycles, 72 ℃ extended 5 min, 4℃-for preservation.
